# Role of acid sphingomyelinase-induced ceramide generation in response to radiation

**DOI:** 10.18632/oncotarget.26526

**Published:** 2019-01-01

**Authors:** Deepa Sharma, Gregory J. Czarnota

**Affiliations:** Gregory J. Czarnota: Department of Radiation Oncology, Sunnybrook Health Sciences Centre, Toronto, Ontario, Canada; Physical Sciences, Sunnybrook Research Institute, Toronto, Ontario, Canada; Department of Medical Biophysics, Faculty of Medicine, University of Toronto, Toronto, Ontario, Canada; Department of Radiation Oncology, Faculty of Medicine, University of Toronto, Toronto, Ontario, Canada

**Keywords:** acid sphingomyelinase, apoptosis, ceramide, microbubbles, ultrasound

Radiation-induced acid sphingomyelinase (*asmase*) stimulation and subsequent ceramide generation are known to be involved in endothelial cell apoptosis. The molecular mechanisms regulating the endothelial response to high doses of radiation are suggested to be initiated *via* translocation of endothelial *asmase* from the inner to the outer leaflet of the plasma membrane. Further, *asmase* catalyzes the hydrolysis of sphingomyelin to generate the lipid second messenger ceramide that leads to the transmembrane signaling of apoptosis [1]. Studies by Garcia-Barros *et al.* demonstrated that mouse MCA/129 fibrosarcoma and B16F1 melanoma upon exposure to single doses of 15-20 Gray (Gy) resulted in *asmase*/ceramide-induced endothelial apoptosis leading to tumour cure. Conversely, tumours xenografted in apoptosis-resistant *asmase* knockout mice were completely resistant to the same single-dose irradiation [2].

Endothelial cells are known to be more susceptible to radiation-induced apoptosis regulated by *asmase* activation because they exhibit an estimated 20-fold higher level of secretory *asmase* relative to other cell types [3][4][5]. Studies have confirmed that endothelial apoptosis occurs significantly after a single high dose of irradiation (>8-10 Gy) *via* an *asmase*-induced ceramide-dependent mechanism, whereas low-dose (1.8-3 Gy) fractionated radiotherapy induces endothelial cell damage involving death signaling pathways [4][6]. The mechanism of endothelial cell apoptosis *via* the *asmase* pathway has been extensively reported by Kolesnick and Fuks. It was demonstrated that upon exposure to radiation, a dose- and time-dependent apoptosis induction was detected in the central nervous system (CNS) of C57BL/6 mice starting from 4 hours and peaking at 12 hours. The study further verified that approximately 20% of the cells undergoing apoptosis were endothelial cells. However, treating mice with basic fibroblast growth factor (bFGF) that acts as an intravascular survival factor for endothelial cells abrogated the cell death process. Similarly, the CNS of *asmase* knockout mice remained negative for cell death staining even after 40 Gy of irradiation [7]. The prior work of Paris *et al.* (2001), demonstrated that whole-body irradiation of C57Bl/6 mice resulted in microvascular endothelial apoptosis that led to the death of animals within 6-8 days from radiation-induced gastrointestinal (GI) syndrome and depleted bone marrow. A close association was found between extensive endothelial apoptosis with that of animal’s death from bone marrow depletion to GI death. In contrast, *asmase* deficient mice or animals treated with bFGF remained abrupt from radiation-induced apoptosis of endothelial cells in the GI tract [8]. Thus, the data confirmed the importance of *asmase* expression for endothelial apoptosis for GI tract dysfunction upon radiation [8].

Several studies have now confirmed radiation-induced *asmase* activation causes vascular collapse followed by endothelial apoptosis to be mandatory for tumour growth inhibition. Contrarily pretreating endothelial cells with the ceramide antagonist sphingosine-1-phosphate (S1P), reverses the *asmase*/ceramide-induced cell death process [9]. Multiple preclinical studies by Czarnota and group have shown that radiotherapy when combined with ultrasound-stimulated microbubbles (USMB) resulted in enhanced therapy response. USMB are recognized to mechanically perturb endothelial cell membranes resulting in a synergistic increase in ceramide-induced vascular disruption and endothelial cell apoptosis [10].

In a recent study published in the Journal of the National Cancer Institute, El Kaffas *et al.* investigated the role of *asmase*-ceramide mechanotransduction by combining USMB with radiation (2 and 8 Gy) *in vivo*. Experiments were conducted in *asmase* wild-type, as well as chemically treated (SIP) and genetically modified endothelial apoptosis-resistant mice (*asmase* knockout) implanted with fibrosarcoma xenografts. Tumour response to treatment was monitored using quantitative 3D Doppler ultrasound and immunohistochemistry at 0, 3, 24, and 72 hours after treatment. Results from experiments with *asmase* wild-type mice, evaluated 24 hours after treatments demonstrated a decrease in tumour perfusion of up to 46% by three hours following radiation and USMB. The drop in tumour perfusion persisted for up to 72 hours and hence was directly linked to an increase in tumour cell death by 53% followed combination of radiation and USMB. In contrary, mice treated with S1P and *asmase* knockout mice revealed no statistically significant decrease in tumour perfusion and no concomitant changes in cell death [11].

El Kaffas *et al.* provide preclinical evidence that the combination of radiation and USMB resulted in significant increases in ceramide generation followed with tumour growth delay in *asmase* wild-type mice. In contrast, S1P-treated or *asmase* knockout mice were reported with minimal ceramide staining and growth delay. It has been confirmed previously that the paradigm of a tumour vascular disruption leading to endothelial apoptosis is directly dependent on ceramide generation [10]. Additionally, the study by El Kaffas *et al.* also reported the changes in the concentration of plasma *asmase* and different ceramide metabolites (ceramide synthase and ceramide kinase). Interestingly, the results revealed high levels of plasma *asmase* and ceramide synthase in *asmase* wild-type mice but not in groups treated with S1P followed radiation and USMB. In opposition, ceramide kinase, an enzyme that metabolizes ceramide to ceramide-1-phosphate was found to be elevated only in *asmase* knockout groups which correlates with the restoration of radioresistance in *asmase* knockout animals [11]. Further, the study provides a rationale for testing dose-dependent radiation effects confirming that a combination of low dose (2 Gy) with USMB produces sufficient ceramide to cause extensive endothelial apoptosis, a phenomenon that was earlier reported only with a higher dose > 8 Gy radiation [4].

To summate, the results of El Kaffas *et al.* study demonstrate for the first time the mechano-acoustic activation of *asmase*/ceramide pathway followed radiation and USMB using genetic and chemical approaches. The paper also highlights some of the limitations of the study. The authors mention that the study lacks the usage of ideal techniques to distinguish between perfused and nonperfused vessels in histopathology. Incorporating perfusion assays as a gold standard histological assessment will help overcome this problem. Secondly, the study addresses the relatively poor resolution of small blood vessel imaging detected by 3D Doppler high-frequency ultrasound and how implementing contrast-enhanced ultrasound or optical imaging could provide with better resolution of blood vessels. Finally, to translate this study in clinical research requires proper planning to maximize the combined effect of radiation and USMB on tumours with minimum effects on healthy tissues. That can be achieved by the selective focussing of ultrasound to only stimulate microbubbles within a tumour target. Collectively, the findings of El Kaffas *et al* provide a basis for understanding the importance of *asmase*/ceramide in radiotherapy and mechanobiology.
